# A Smartphone Platform for Remote Motor Fitness Assessment and AI-Generated Personalized Exercise Programs for Older Adults: Randomized Controlled Trial

**DOI:** 10.2196/73145

**Published:** 2025-10-15

**Authors:** Yael Netz, Salit Bar-Shalom, Esther Argov, Michal Arnon, Eti Benmoha, Ziv Yekutieli, Keren Tchelet Karlinsky, Jeremy M Jacobs

**Affiliations:** 1 Levinsky-Wingate Academic College, Campus Wingate, Wingate Institute, Netanya, 4290200, Israel, +972-9-8639314; 2 Montfort Brain Monitor LTD, Tel Aviv, Israel; 3 Department of Geriatric Rehabilitation and Center for Palliative Care, Hadassah Medical Center and Faculty of Medicine, Hebrew University of Jerusalem, Jerusalem, Israel

**Keywords:** home-based exercise, machine learning, digital markers, dynamic balance, static balance, arm flexibility, arm strength

## Abstract

**Background:**

Exercise guidelines for older adults are predominantly “one-size-fits-all,” primarily focusing on aerobic activity with limited emphasis on motor components.

**Objective:**

We examined the hypothesis that remotely delivered, personalized multicomponent exercise—based on a simple yet highly reliable and accurate smartphone motor fitness assessment and individually tailored using machine learning—can improve balance, flexibility, and strength among older adults, obviating the need for a laboratory or professional supervision.

**Methods:**

This randomized controlled study recruited community-dwelling healthy older adults aged ≥65 years, with normal cognition, low fall risk, and no hospitalization within the last year for cardiac/neurological illness. Participants were randomly assigned to an experimental 8-week personalized exercise group (5×/wk, multicomponent exercises), an 8-week active-control group (exercise counseling according to World Health Organization guidelines), or a control group (no intervention). Participants were assessed at baseline, 4, 8, and 12 weeks. Measurements were remotely recorded using smartphone sensors and analyzed using machine learning to create each participant’s unique fitness profile. Primary outcomes were fitness profile changes at 8 weeks.

**Results:**

We assessed 317 volunteers; 239 of them consented and met inclusion criteria (155 women, mean age 72.63, SD 5.38 y). Compared to both controls, the personalized exercise group significantly improved in dynamic balance (*F*_6,404_=3.232, *P*=.004, η^2^=0.046), total balance (sum of all balance measurements; *F*_6,432_=3.03, *P*=.006, η^2^=0.040), arm flexion (*F*_6,448_=2.527, *P*=.02, η^2^=0.033), arm extension (*F*_6,450_=2.753, *P*=.01, η^2^=0.035), and arm strength (*F*_6,424_=2.394, *P*=.03, η^2^=0.033). Significant improvement was observed with adherence as low as 1.5 exercise sessions/week over 8 weeks and often within just 4 weeks. No improvement was observed on torso rotation and on sit-to-stand.

**Conclusions:**

A smartphone platform, with remote assessment and delivery of home-based individually tailored exercises, effectively targets the often-neglected key fitness components—balance, arm flexibility, and arm strength—in older adults. This approach has the potential to generate varied movement profiles and personalized exercise programs for both healthy individuals and those with mobility or cognitive impairments.

## Introduction

Physical activity is associated with numerous benefits among older people [1-5] and is a key recommendation for promoting healthy aging [6]. Advancing age is associated with increasing diversity and variability across a wide range of biological, physiological, functional, and performance measures—an expression of the growing disparity between biological and chronological age which typifies aging [7,8]. Accordingly, the effectiveness of current exercise (structured, purposeful physical activity) guidelines [9,10], which adopt a “one-size-fits-all” approach, is questionable, with protagonists emphasizing the need for a personalized approach [11].

An additional limitation of current guidelines is the lack of detailed attention concerning fitness components other than cardiovascular fitness [9,10]. While guidelines specify the duration, intensity, and frequency of aerobic exercise, minimal guidance exists concerning optimizing balance, strength, and flexibility—all crucial factors for preserving functional integrity with advancing age. In particular, the absence of accurate individualized assessments of these components outside a laboratory setting hinders the transition from generalized to personalized exercise programs for older adults.

The potential contribution of artificial intelligence in shaping personalized medicine is of current interest, including the promotion of personalized physical activity [12,13]. Nonetheless, it remains a fact that current physical activity guidelines are manually formulated, generic, and focus on group rather than personalized exercises.

To address these limitations, we developed a novel home-based approach to personalized exercise programs for older adults, utilizing a simple smartphone that obviates the need for a laboratory or professional intervention. Through smartphone accelerometer and gyroscope sensors, we remotely assess key components of motor fitness, including balance, flexibility, and strength. Based on these assessments, a machine learning–generated personalized exercise program, tailored to each individual’s needs, was delivered via video directly on the smartphone.

We previously described the design, development, validation, and pilot study results [14-16]. Briefly, an interdisciplinary expert panel selected motor components and standard movement tests for remote fitness assessment. These were incorporated into a user-friendly smartphone app, which provided simple audiovisual instructions for self-testing, recorded the test results, and uploaded the raw data to a remote study database, where machine learning was used to create a unique fitness profile for each participant. In order to create personalized exercise programs, we developed a collection of exercises specifically designed for older adults, spanning the different movement abilities (balance, flexibility, and strength) and graded according to difficulty. According to the participants’ unique fitness profile, a tailored selection of exercises was chosen using machine learning, uploaded to the app, and remotely delivered to the study participant via the study app. With repeated fitness testing and ongoing data collection, the precision of machine learning for matching fitness profile to tailored exercise programs is constantly improving.

In this study, we tested the implementation of our approach in a randomized controlled trial. Our objective was to investigate the effectiveness of an 8-week, remotely delivered personalized exercise program based on individual fitness assessments, compared to either WHO general guidelines (active-control) or no intervention (control). We hypothesized that participants in the personalized experimental group would show greater improvements in balance, flexibility, and strength than those in the active-control and control groups. Specifically, we anticipated a reduction in body sway during balance tests, an increased range of motion in flexibility, and faster lifts of the body or arms in strength. The real-life implications and subsequent benefits of improvements across this range of motor fitness components are notable. Thus, for example, well-established evidence highlights that balance improvement is critical for fall prevention [1,17,18], while flexibility [19,20] and strength [21,22] are essential for performing activities of daily living.

## Methods

### Study Design

A randomized controlled trial was conducted, with participants randomly assigned to three groups: (1) experimental group (personalized exercise); (2) active-control group (World Health Organization [WHO] general exercise guidelines); and (3) control group (no intervention).

### Sample Size Predetermination

Based on a statistical power analysis using G*Power [23], our target was to enroll 300 participants, with 100 in each of the 3 treatment groups (see more details in our protocol paper [14]). However, to maintain methodological rigor, we later conducted a more precise power analysis based on the actual number of participants included in the statistical analyses (see Results section).

### Participants

The study was conducted in a community setting (private homes and independent living facilities) in central and northern Israel, including both urban and agricultural settlements. Recruitment included local flyers and lectures delivered by the principal investigator in community clubs, senior centers, cultural centers, or other local gathering places. Participant enrollment was carried out by the study team, which consisted of 6 testers and a study manager.

From a total of 317 volunteers, 239 community-dwelling, healthy older adults (155 women), aged 72.63 (SD 5.38) years, completed the 8-week study intervention, and 230 completed the full 12-week follow-up. Inclusion criteria were (1) age 65+ years, (2) independent living, (3) independent walking, (4) fluent in Hebrew, and (5) smartphone competency. Exclusion criteria included (1) cognitive impairment (Mini-Cog score <3) [24], (2) any hospitalization (>24 h) or emergency room visit within past year for cardiac (heart failure, rhythm disorder, ischemia, valvular disease) or neurological conditions (dizziness, cerebrovascular, vestibular, progressive diseases affecting gait or balance), and (3) high fall risk (≥1 positive answer from 3-item test), validated in community-based exercise intervention and fall-prevention studies [25]. Data collection included demographics, self-reported habitual physical activity, depression status (Geriatric Depression Scale) [26], and frailty index [27].

### Ethical Considerations

The study design, procedures, and informed consent were approved by the Hadassah Hebrew University Hospital Ethics Committee, Jerusalem, Israel (Trial ID 0074‐19-HMO), and were conducted according to the ethical standards of the Helsinki Declaration.

#### Informed Consent

Written informed consent was attained following a detailed explanation given by the study physician and included instructions on how to act in case of any possible medical complications arising either directly (eg, risk of falls and fractures, musculoskeletal pains, strained ligaments) or secondary to the exercise programs (eg, cardiac arrhythmias or angina). Participants were informed of their freedom to opt out.

#### Privacy and Confidentiality

Each participant was assigned a unique study identification number. The key linking these numbers to participant identities was stored separately by the principal investigator. All data were subsequently deidentified.

#### Compensation Details

Participants did not receive any compensation.

### Randomization

We used a permuted block design [7] with 2 stratification factors (age: 65‐74 and 75+ years; gender: men and women), each block included 6 participants, 2 per group. Block distribution was adjusted using the minimization principle [28] in order to address randomization imbalance. More specifically, as there were 6 testers, and each tester had his or her randomization table. This process was supervised by an advanced student (EB).

At a certain point, we realized that few testers had the first participants on the control group, and the control group became a lot larger than the other groups. Based on the minimization principle, we changed the block distribution to 2 experimental, 2 active-control, and only 1 control (5 participants in a block).

### Outcome Measures

The motor components chosen for remote fitness assessment were postural control (stability) while standing (static balance) or moving (dynamic balance), strength (muscle endurance) of upper and lower body, and range of motion of upper body (flexibility). The following standard movement performance tests were selected to assess these components:

1. Static balance: Leg stance (single leg stance left and right) for 10 seconds.
2. Static balance: Tandem stance—one foot directly in front of the other (left foot forward and right foot forward) for 20 seconds.
3. Dynamic balance: Tandem walk forward (10 steps) and tandem walk backward (10 steps).
4. Upper body flexibility (torso rotation): Seated position, holding a ball between thighs and baton in hands in front of chest for stabilization [29]; maximal torso rotation to left and right.
5. Upper body flexibility (arm flexion): Seated position, armless chair, back against wall; lifting straight arm (right and left separately) forward and up, trying to reach the ear.
6. Upper body flexibility (arm extension): Standing position, face towards wall, all front body against wall; lifting straight arm (right and left separately) backward as far as possible.
7. Upper body strength (arm strength): Seated position on an armless chair, a 0.5 kg weight for women and 1 kg for men attached to wrist; lifting straight arm (left and right separately) forward up to shoulder height as fast as possible 20 times and to the side 20 times.
8. Lower body strength (sit-to-stand): From sitting position, hands on waist, stand and sit 10 repetitions.

**Figure 1.**
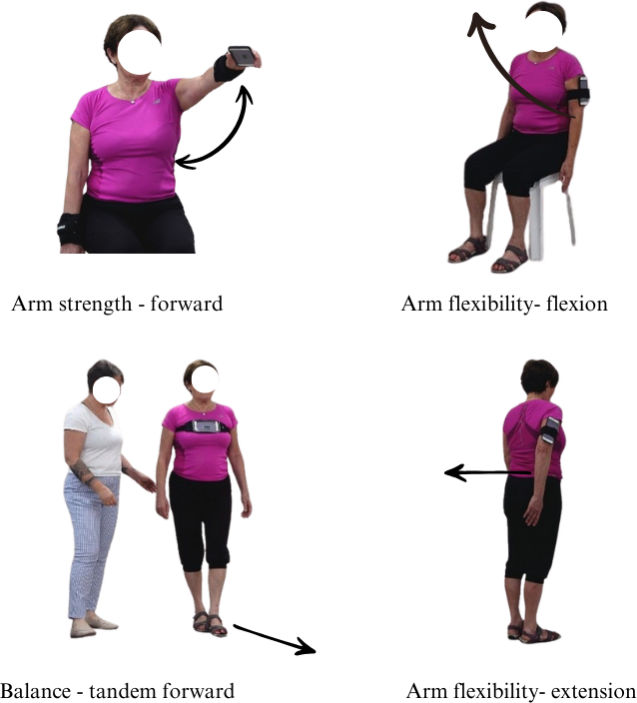
Examples of phone placement in tandem forward, arm flexion, arm extension, and arm strength.

Instructions were incorporated into a smartphone app developed by Montfort Brain Monitor Ltd. Movement was captured by smartphone accelerometer and gyroscope sensors by attaching the mobile phone to the relevant body part using a simple band (see Figure 1: tandem walk forward, arm flexion, arm extension, arm strength).

### Digital Markers

The specific digital markers (DMs) generated from the phone for each test are as follows:

- Balance tests (Tests 1‐3): Five DMs that assess body sway, with lower scores indicating less sway and better performance:
  1. Average linear acceleration (m/s²), generated by the accelerometer in the mediolateral direction.
  2. Average linear acceleration (m/s²), generated by the accelerometer in the anterior-posterior direction.
  3. Angular (radial) velocity, assessed in radians per second (rad/s), generated by the gyroscope in the mediolateral direction.
  4. Angular (radial) velocity, assessed in radians per second (rad/s), generated by the gyroscope in the anterior-posterior direction.
  5. Angular (radial) velocity, assessed in radians per second (rad/s), generated by the gyroscope in the superior-inferior direction.

- Torso rotation (Test 4): The angle (peak pitch) in the superior-inferior direction. A greater angle indicates a larger range of motion and better performance.
- Arm flexion (Test 5): The angle between the arm and the horizon (peak yaw in the anterior-posterior direction).
- Arm extension (Test 6): The angle between the arm and the horizon (peak yaw in the anterior-posterior direction). A greater angle indicates a larger range of motion and better performance.
- Arm strength (Test 7): Average duration for 20 repetitions, measured in seconds. A shorter duration indicates better performance.
- Sit-to-stand (Test 8): Average duration for each repetition, measured in seconds. A shorter duration indicates better performance.

Detailed information and graphical demonstrations of the DMs have been previously published [14,15].

### Study Procedure

Data collection started in November 2020 and ended in September 2023. Following informed consent, demographic/clinical data collection, and randomization, baseline fitness (T0) was assessed using the study app. Fitness levels across the study measures were determined (low versus high), and assessment was repeated at 4 weeks (T1), 8 weeks (T2)*,* and 12 weeks (follow-up T3). The assessment was conducted by 6 qualified physical activity teachers, each trained to use the app for testing participants individually and supervised by the study manager, a senior physical activity teacher. The app was installed on the teachers’ (testers’) smartphones, and the test results were automatically streamed to the database. All study participants (the personalized exercise group, the active-control group, and the control group) were tested. The testers and participants were blinded to the test results. Participants received weekly phone calls from the teachers to maintain contact and motivation. There were no issues of safety or other issues reported.

### Intervention Groups

#### Personalized Exercise (Experimental Group)

**Figure 2.**
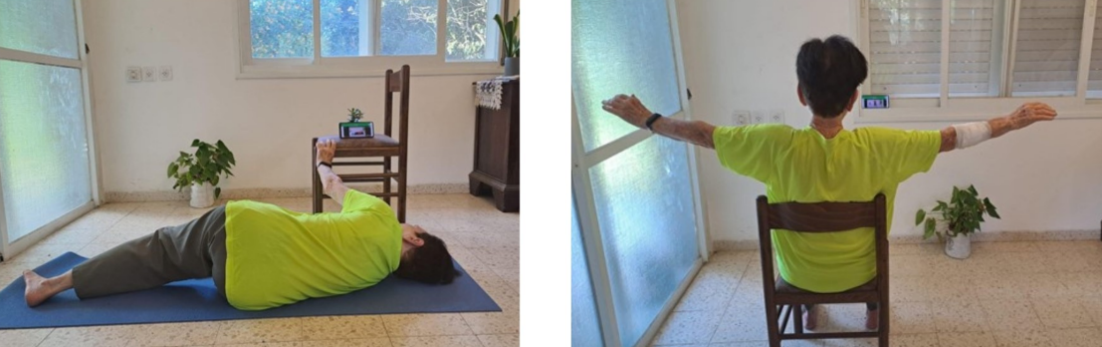
Practicing at home with the exercise displayed on the phone.

Participants in the experimental group received their personalized exercise program based on their fitness assessment. The personalized program, including clear instructions regarding the performance of the exercises, was delivered to the participants’ personal smartphones immediately after the testing. The testers explained to the participants, on an individual basis, how to use the app on their personal smartphones for exercising. Following our pilot study [15], and according to evidence concerning the advantages of exercising >3×/week [9,10], we instructed participants to exercise 5×/week for 8 weeks. Their video exercise program covered three target categories: (1) balance and lower body, (2) upper body flexibility, and (3) upper body strength exercises. Two difficulty levels, A (simple) and B (advanced), were matched according to fitness assessment. For an example of exercise displayed on a smartphone, please see Figure 2. Additional examples have been described previously [14].

#### General Exercise (Active Control Group)

Participants were individually counseled and advised to exercise for 8 weeks according to official WHO guidelines [9]. Specifically, they were asked to perform leisure-type aerobic exercise for 150‐300 minutes or vigorous-intensity exercise for 75‐150 minutes/week. Examples such as walking, jogging, and cycling were given. In addition, they were asked to perform ≥3 sessions/week of multicomponent physical activity that emphasizes functional balance and strength training at moderate or greater intensity. They received the following examples of balance exercises: standing on toes, one-leg stance, walking while lifting the knee, walking backward, and side walking while bending and extending the knees. Examples of strength exercises included the following: (1) in a standing position—lifting the leg to the side (abduction), extending a straight leg backward, lifting a straight leg forward, sit-to-stand movements—and (2) against a wall—pushing the body away from the wall using the hands, lifting the arms to the side, optionally with dumbbells.

#### Control Group

Participants were advised to continue their normal routine. A personalized exercise program was offered after study completion.

### Matching Fitness Level With Exercise Prescription for the Personalized Exercise Group—A Machine Learning Approach

**Figure 3.**
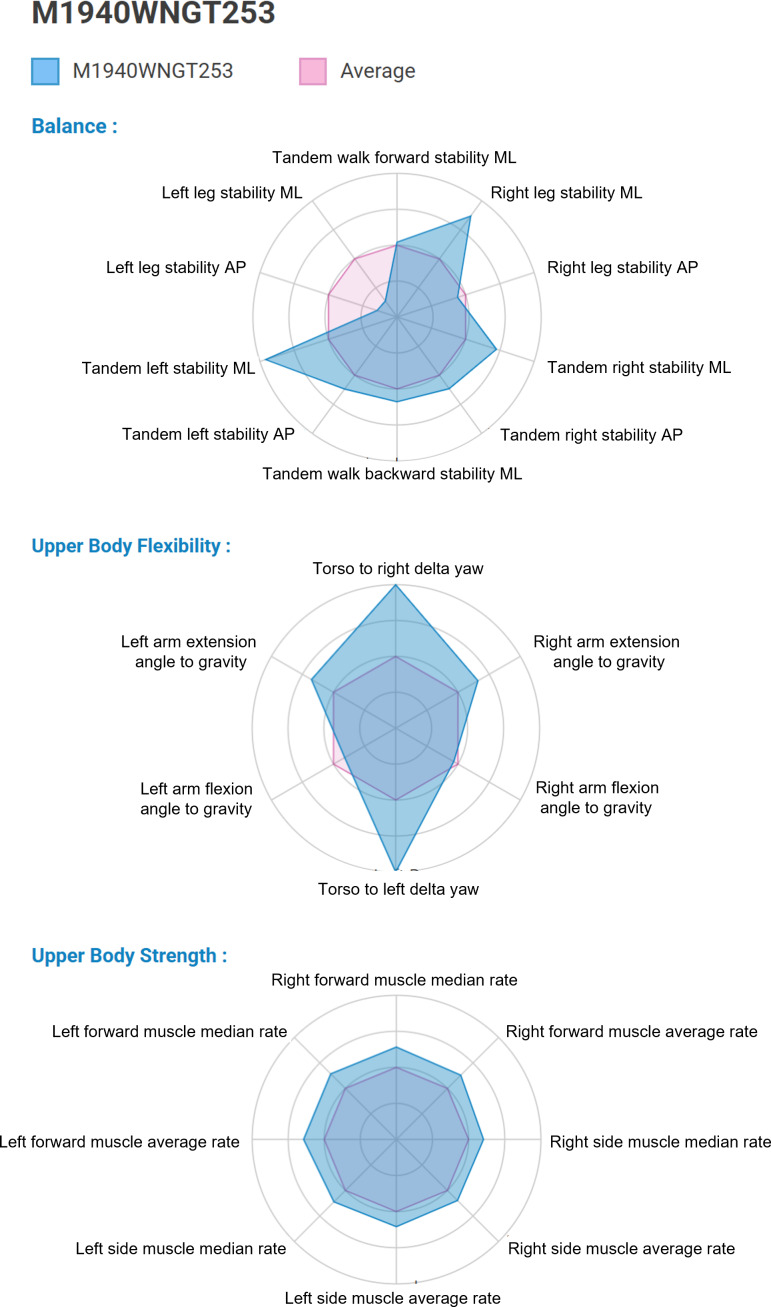
A participant’s unique fitness profile graphically displayed alongside the average profile. AP: anterior-posterior; ML: mediolateral.

Data from the pilot study [15] served as a baseline for large-scale data collection, and the baseline (T0) fitness level of participants in the current study (low or high) was determined using machine learning principles. Based on the fitness level, the app determined the appropriate level of exercise difficulty (A or B) for each fitness component. For example, a participant in the personalized exercise group might have been assigned level A for balance exercises, level B for flexibility, and level B for strength. Figure 3 illustrates an example of a study participant’s unique fitness profile, based on the DMs, graphically displayed alongside the average profile for the entire study sample. Repeated fitness assessments at 4 weeks (T1) allowed for the personalized exercise program to be adjusted according to the updated DMs.

### Data Transformation

For comparability reasons due to different units of measurement, we transformed the DM data into normalized (*z*) scores. For balance measures, we calculated the mean of the 5 generated DM scores to one for each balance test. Results are presented as *z* scores.

### Adherence to Program Recommendations: Adherers and Nonadherers in the Personalized Exercise Group—Post Hoc Distribution

Since participants needed to access the study app in order to watch and perform exercise videos, it was therefore possible to determine the actual and accurate measurement of adherence. Although they were instructed to exercise 5×/week, the actual adherence varied. To explore whether adherence influenced improvements, we conducted an exploratory analysis categorizing participants as adherers or nonadherers. The following cutoff points, along with their rationale, were examined:

- Stage 1: ≥3 sessions/week versus <3 sessions/week (based on official recommendations, whereby 3 sessions/week is considered optimal) [9].
- Stage 2: ≥2.45 sessions/week versus <2.45 sessions/week (the median adherence score).
- Stage 3: ≥2 sessions/week versus <2 sessions/week (a lower threshold to determine the minimum frequency needed for fitness improvements).
- Stage 4: ≥1.5 sessions/week versus <1.5 sessions/week (an even lower threshold to determine the minimum frequency needed for fitness improvements).

A consistent trend favoring adherers over nonadherers emerged across all cutoff points (see Multimedia Appendix 1). The most notable differences were observed with the ≥2 sessions/week versus <2 sessions/week and ≥1.5 sessions/week versus <1.5 sessions/week cutoffs.

Ultimately, we selected 1.5 sessions/week as the final cut-off for two reasons:

1. This frequency suggested that significant improvements in balance, flexibility, and strength could be achieved with as few as 1.5 tailored exercise sessions per week.
2. It served as a criterion for distinguishing nonexercisers while still retaining a sizable proportion of participants (71.7%; see Multimedia Appendix 1) in the personalized exercise group.

Subsequently, we conducted statistical analyses using four groups: (1) personalized exercise adherers (n=66), (2) personalized exercise nonadherers (n=26), (3) general guidelines exercise (active-control; n=80), and (4) control (n=67).

### Statistical Analyses

We applied statistical analyses to the 3-time measurements during the intervention period, T0, T1, and T2, and examined whether improvements observed after 8 weeks (T2) were maintained at 12-week follow-up (T3). Specifically, a mixed repeated measures ANOVA (3 test dates×4 groups) was conducted for each fitness component, and Eta squared (η²) was calculated to assess the effect size. Fisher LSD was used for pairwise post hoc analyses, and Cohen *d* coefficients were calculated to reveal the standardized differences between means when effects reached a significance level (*P*<.05).

To examine whether improvements were maintained at T3, we examined participants from the general group and personalized adherers who demonstrated improvement from T0 to T2 (between the baseline and the end of the 8-week intervention period). Improvement was defined as a change of ≥0.12 in *z* score (*z*T2-*z*T0), corresponding to the 55th percentile with normal distribution. More specifically, individuals with T2-T0 *z* scores ≥0.12 were considered “improvers.” Two-way ANOVA (2 test dates×2 groups) with repeated measures was applied on the outcome measures.

### Attrition Rate (Percentage of Remaining Participants in Each Group)

Due to incomplete compliance during 1 or more measurements, or failure to upload measurements due to poor internet connectivity, specific data points for certain individuals were excluded. Additionally, scores greater than 2.5 SDs from the raw data means were omitted. As a result, the number of participants in each ANOVA (assessing the effect of the intervention on each fitness component) ranged from 189 to 231. The participation rate (%) for each group was calculated for the main outcomes (see Multimedia Appendix 2). *χ*² analyses revealed no significant differences. The power calculation was based on a sample size of n=189.

## Results

### Participants

**Figure 4.**
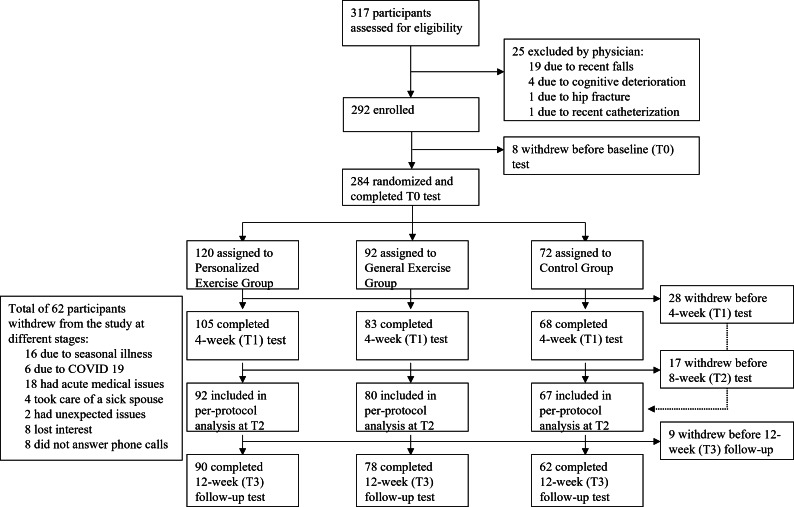
Participants’ flowchart.

Figure 4 illustrates the participant flowchart across the 3 original study groups. Of those who completed the 12 weeks, 90 were from the personalized exercise group, 78 from the general exercise group, and 62 from the control group.

Table 1 presents baseline data.

**Table 1.** Baseline characteristics.

	Personalized exercise (n=92)	General exercise (n=80)	Control (n=67)
Female, n (%)	55 (59.7)	57 (71.3)	43 (64.2)
Male, n (%)	37 (40.2)	23 (28.8)	24 (35.8)
Age (y), mean (SD)	72.37 (5.01)	72.80 (5.58)	72.78 (5.70)
Height (cm)			
Female, mean (SD)	161.92 (5.68)	160.84 (6.20)	161.90 (5.64)
Male, mean (SD)	172.22 (6.00)	173.48 (5.16)	175.22 (4.30)
Weight (kg)			
Female, mean (SD)	68.42 (10.73)	69.65 (12.93)	69.02 (10.00)
Male, mean (SD)	78.97 (12.68)	83.3 (10.75)	80.17 (6.34)
Working			
No, n (%)	36 (39.1)	26 (35.6)	31 (47.7)
Yes, n (%)	29 (31.5)	21 (28.8)	20 (30.8)
Volunteer, n (%)	23 (25.0)	26 (35.6)	14 (21.5)
Married/living with a partner, n (%)	66 (71.7)	53 (66.3)	47 (70.1)
Don’t smoke, n (%)	86 (93.5)	73 (91.3)	63 (94.0)
Score 0 on Short GDS^a^, n (%)	89 (96.7)	78 (97.5)	62 (96.9)
Frailty (out of 41 deficits), mean (SD)	2.85 (1.92)^b^	3.64 (2.14)^b^	3.13 (2.08)
Engaged in physical activity during the last 7 d, n (%)	79 (85.9)	61 (76.3)	56 (83.6)
Aerobic activity in the last 7 d (min), mean (SD)	227.28 (247.04)	173.63 (185.08)	232.84 (227.19)
Other exercise in the last 7 d (min), mean (SD)	87.22 (98.07)	76.50 (89.73)	77.46 (115.80)
Total exercise last 7 d (min), mean (SD)	314.51 (256.86)	250.13 (212.59)	310.30 (275.55)
Sedentary time during 1 d (h), mean (SD)	6.58 (4.26)	5.69 (3.41)	6.82 (4.57)
Active in the last 7 d (1‐5 scale), mean (SD)	4.03 (0.93)	4.04 (0.91)	4.30 (0.65)

a GDS: Geriatric Depression Scale.

b *P*<.05

### Power Analysis Based on the Post Hoc Distribution (Four Groups)

We used G*Power [30] to perform power analysis for a 2-way ANOVA (3 test dates×4 groups) with repeated measures on the outcome measures. Our sample (n=189) provided 91% statistical power to find an interaction with small effect (Cohen *f*=0.1), 91% power to find group differences of moderate effect (Cohen *f*=0.25), and 97% power to find within measurements differences of small effect (Cohen *f*=0.1). All power analyses used a correlation of 0.685 among repeated measures because, among all our primary outcomes, this was the lowest (the highest was 0.856) and thus the most conservative value to use.

### Results of the Statistical Analyses

**Figure 5.**
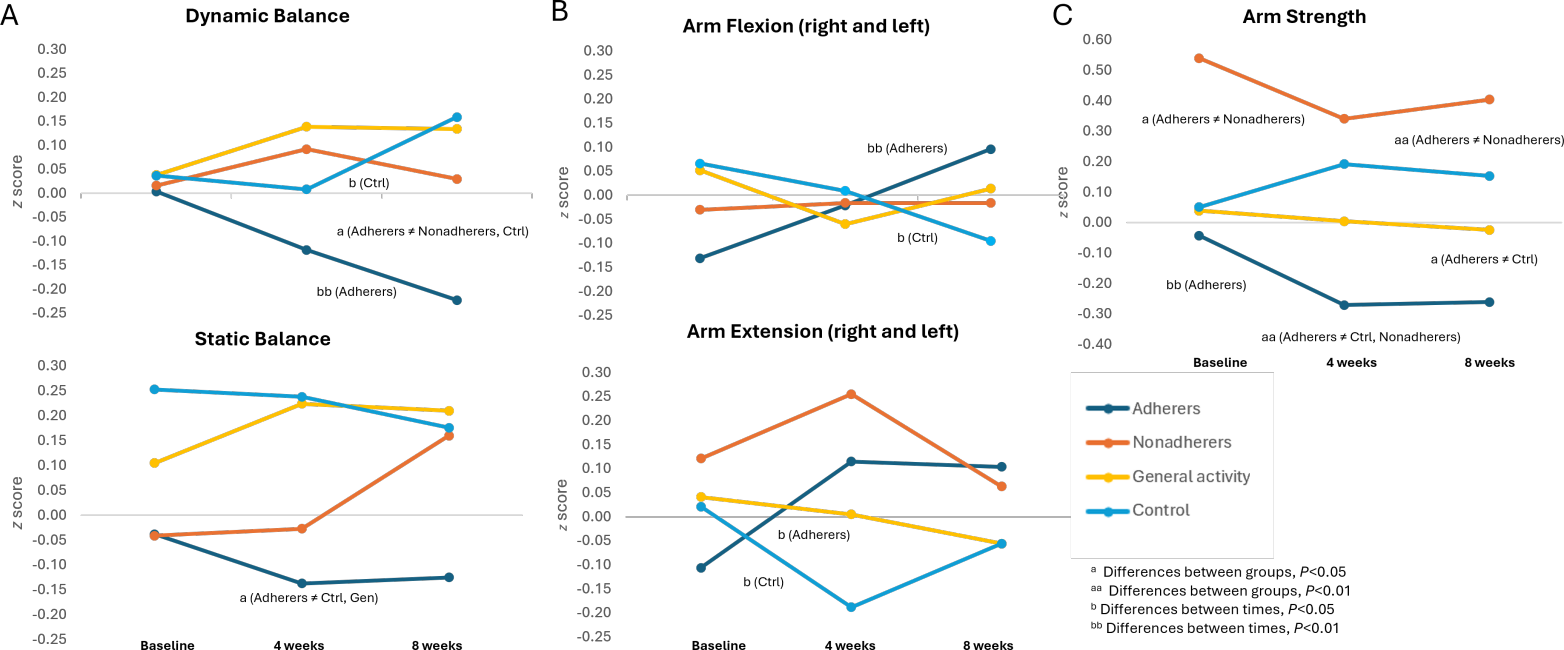
*z* scores of (A) dynamic and static balance, (B) arm flexion and extension, and (C) arm strength. Ctrl: control.

Figure 5 describes the main results. Group×time interaction was significant for dynamic balance (mean tandem walk forward and backward, *F*_6,404_=3.232, *P*=.004, η^2^=0.046; Figure 5A). Pairwise analyses indicated significant improvements among personalized exercise adherers (Adherers) from T0 to T2 (*M*_diff0,2_=0.228, *P*=.002, *d*=0.404) and group differences in favor of the Adherers in T2 (*M*_grp1,3_=−0.357, *P*=.02, *d*=0.456; *M*_grp1,4_=−0.383, *P*=.01, d=0.474; Figure 5A). The interaction on static balance (mean of leg stance and tandem stance) was not significant, but group differences favored the Adherers at T1 (*M*_grp1,3_=−0.375, *P*=.01, *d*=0.430; *M*_grp1,4_=−0.361, *P*=.02, *d*=0.446; Figure 5A).

Group×time interactions were revealed on both arm flexion (mean right and left, *F*_6,448_=2.527, *P*=.02, η^2^=0.033) and arm extension (mean right and left, *F*_6,450_=2.753, *P*=.01, η^2^=0.035; Figure 5B). Follow-up pairwise analyses indicated significant improvement in the Adherers on arm flexion (left and right) from T0 to T2 (*M*_diff0,2_=−0.227, *P*=.007, *d*=0.356) and on arm extension (left and right) from T0 to T1 (*M*_diff0,1_=−0.221, *P*=.02, *d*=0.302) and from T0 to T2 (*M*_diff0,2_=−0.210, *P*=.03, *d*=0.290; Figure 5B).

Group×time interaction was also indicated on arm strength total (mean of lifting right arm forward, left arm forward, left arm to the side, and right arm to the side; *F*_6,424_=2.394, *P*=.03, η^2^=0.033; Figure 5C). Follow-up pairwise analyses indicated significant improvement among the Adherers on arm strength total from T0 to T1 (*M*_diff0,1_=0.228, *P*=.008, *d*=0.438) and from T0 to T2 (*M*_diff0,2_=0.217, *P*=.008, *d*=0.384; Figure 5C).

More results are presented in the tables below. Regarding balance, group×time interactions were significant for tandem stance left (left foot forward, η^2^=0.033), tandem walk forward (η^2^=0.039), tandem walk backward (η^2^=0.034), and balance total (mean of all balance scores, η^2^=0.040; Table 2). Pairwise analyses indicated improvements (time differences) only in the Adherers from T0 to T2 (*M*_diff0,2_=0.163, *P*=.009, *d*=0.259), with group differences favoring the Adherers at T1 (*M*_grp1,3_=0.344, *P*=.007, *d*=0.470) and at T2 (*M*_grp1,3_=0.399, *P*=.003, *d*=0.520, *M*_grp1,4_=0.347, *P*=.01, *d*=0.480; Table 2).

Regarding flexibility, group×time interactions were revealed on right arm flexion (η^2^=0.038), right arm extension (η^2^=0.032), left arm (mean flexion and extension, η^2^=0.035), and right arm (mean flexion and extension, η^2^=0.054; Table 3). Pairwise analyses indicated significant improvements only among the Adherers on right arm flexion from T0 to T2 (*M*_diff0,2_=−0.321, *P*=.002, *d*=0.425), on right arm extension from T0 to T1 (*M*_diff0,1_=−0.264, *P*=.02, *d*=0.293), and from T0 to T2 (*M*_diff0,2_=−0.262 , *P*=.03, *d*=0.309), on right arm (mean flexion and extension) from T0 to T1 (*M*_diff0,1_=−0.205, *P*=.008, *d*=0.341) and from T0 to T2 (*M*_diff0,2_=−0.283, *P*<.001, *d*=0.625), and on left arm (mean flexion and extension) from T0 to T1 (*M*_diff0,1_=−0.192, *P*=.009, *d*=0.359; Table 3). No change was observed on torso rotation (Table 3).

**Table 2.** *z* scores of T0 (baseline), T1 (4 wk), and T2 (8 wk) balance measures.

	Personalized exercise adherers^a^ (1)	Personalized exercise nonadherers^b^ (2)	General activity (3)	Control (4)	*F* Time (*df*)	*F* Group (*df*)	*F* Interaction (*df*)
Left leg stance, mean (SD)					0.65 (2, 336)	1.91 (3, 168)	1.24 (6, 336)
T0	−0.189 (0.914)	−0.469^c^ (0.419)	−0.136 (0.719)	0.221^c^ (1.089)			
T1	−0.161 (0.892)	−0.150 (0.670)	0.064 (1.006)	0.020 (0.968)			
T2	−0.218 (0.762)	−0.205 (0.837)	0.009 (0.830)	0.087 (1.096)			
Right leg stance, mean (SD)					0.27 (2, 340)	3.54^d^ (3, 170)	0.55 (6, 340)
T0	−0.193 (0.762)	−0.386 (0.678)	0.031 (0.889)	0.012 (0.935)			
T1	−0.251^c^ (0.590)	−0.288 (0.721)	0.079 (1.049)	0.136^c^ (1.084)			
T2	−0.313^e^ (0.585)	−0.346 (0.542)	0.015 (0.853)	0.192^e^ (1.108)			
Tandem stance left, mean (SD)					1.01 (2, 404)	2.91^d^ (3, 202)	2.32^d^ (6, 404)
T0	−0.119 (0.708)	−0.019 (1.053)	−0.100 (0.746)	0.181 (1.069)			
T1	−0.366^e^^,^^f^ (0.599)	−0.159 (0.816)	0.062 (0.842)	0.179^e^ (0.987)			
T2	−0.255^c^ (0.792)	0.115 (1.208)	0.107^c^^,^^g^ (0.967)	0.084^c^ (0.781)			
Tandem stance right, mean (SD)					0.19 (2, 380)	1.22 (3, 190)	1.04 (6, 380)
T0	−0.030 (0.806)	−0.092 (1.020)	−0.164 (0.779)	0.134 (0.929)			
T1	−0.184 (0.990)	−0.215 (0.756)	0.041 (0.940)	0.120 (0.886)			
T2	−0.131 (0.975)	0.001 (1.209)	−0.061 (0.838)	0.128 (0.725)			
Tandem walk forward, mean (SD)					0.13 (2, 380)	1.05 (3, 190)	2.56^d^ (6, 380)
T0	−0.009 (0.875)	−0.111 (0.941)	0.037 (0.924)	0.082 (0.899)			
T1	−0.118 (0.809)	−0.023 (0.981)	0.164 (0.929)	−0.035 (0.925)			
T2	−0.266^c^^,^^h^ (0.704)	−0.083 (0.962)	0.146^c^ (0.948)	0.105^c^ (0.873)			
Tandem walk backward, mean (SD)					0.01 (2, 382)	0.85 (3, 191)	2.21^d^ (6, 382)
T0	−0.007 (0.750)	0.064 (1.092)	0.033 (0.844)	−0.033 (0.920)			
T1	−0.143^c^ (0.881)	0.030 (0.837)	0.191^c^ (0.915)	0.011 (0.841)			
T2	−0.162^c^ (0.810)	−0.068 (1.002)	0.143 (0.856)	0.176^c^^,^^g^ (0.985)			
Leg stance (L+R), mean (SD)					0.04 (2, 370)	2.66^d^ (3, 185)	0.54 (6, 370)
T0	−0.138^c^ (0.776)	−0.259^c^ (0.786)	0.093 (0.899)	0.245 (1.019)			
T1	−0.148 (0.758)	−0.155 (0.600)	0.149 (0.993)	0.133 (0.948)			
T2	−0.231^c^ (0.687)	−0.110^c^ (0.905)	0.080^c^ (0.878)	0.176^c^ (0.986)			
Tandem stance (L+R), mean (SD)					0.30 (2, 420)	2.00 (3, 210)	2.11^i^ (6, 420)
T0	−0.022 (0.725)	0.008 (0.916)	−0.048 (0.772)	0.218 (1.000)			
T1	−0.214^e^^,^^f^ (0.760)	−0.092 (0.830)	0.101^c^ (0.822)	0.262^e^ (0.961)			
T2	−0.143 (0.933)	0.115 (1.099)	0.096 (0.872)	0.150 (0.741)			
Balance total, mean (SD)					0.51 (2, 432)	2.11 (3, 216)	3.03^d^ (6, 432)
T0	−0.019 (0.641)	−0.010 (0.786)	0.102 (0.783)	0.128 (0.801)			
T1	−0.123^c^^,^^f^ (0.642)	0.068 (0.719)	0.221^c^^,^^f^ (0.795)	0.120 (0.732)			
T2	-0.182^c^^,^^g^ (0.712)	0.132 (0.921)	0.217^c^^,^^g^ (0.811)	0.165^c^ (0.721)			

a Adherers: ≥1.5/wk

b Nonadherers: <1.5/wk

c Differences between groups (*P*<.05).

d *P*<.05.

e Differences between groups (*P*<.01).

f Differences between T0 and T1 (*P*<.05).

g Differences between T0 and T2 (*P*<.05).

h Differences between T0 and T2 (*P*<.01).

i *P*=.05.

**Table 3.** *z* scores of T0 (baseline), T1 (4 wk), and T2 (8 wk) flexibility measures.

	Personalized exercise adherers^a^ (1)	Personalized exercise nonadherers^b^ (2)	General activity (3)	Control (4)	*F* Time (*df*)	*F* Group (*df*)	*F* Interaction (*df*)
Left arm flexion, mean (SD)					0.29 (2, 434)	0.09 (3, 217)	1.30 (6, 434)
T0	–0.132 (0.959)	–0.115 (1.031)	0.052 (0.990)	0.074 (1.005)			
T1	0.026 (0.978)	–0.091 (0.929)	–0.066 (0.982)	0.007 (1.000)			
T2	0.032 (0.968)	–0.032 (0.959)	0.059 (1.088)	–0.032 (0.870)			
Right arm flexion, mean (SD)					0.08 (2, 420)	0.07 (3, 210)	2.78^c^ (6, 420)
T0	–0.175 (0.946)	–0.009 (0.887)	0.067 (0.899)	0.062 (1.160)			
T1	–0.054 (0.976)	–0.063 (0.985)	0.002 (0.963)	0.018 (1.023)			
T2	0.146^d^^,^^e^ (1.059)	–0.035 (1.080)	0.025 (1.014)	–0.145^d^ (0.909)			
Left arm extension, mean (SD)					0.01 (2, 434)	0.99 (3, 217)	1.95 (6, 434)
T0	–0.001 (0.989)	0.039 (1.036)	0.013 (0.99)	–0.021 (1.029)			
T1	0.138^f^ (0.963)	0.168^f^ (0.845)	0.050^f^ (0.932)	–0.315^f^^,^^g^ (1.029)			
T2	0.117 (0.883)	0.105 (0.959)	–0.044 (1.055)	–0.129 (0.883)			
Right arm extension, mean (SD)					0.27 (2, 432)	0.51 (3, 216)	2.40^c^ (6, 432)
T0	–0.122 (1.004)	0.156 (0.768)	0.068 (1.025)	–0.037 (1.007)			
T1	0.142^h^ (0.970)	0.239 (0.871)	–0.032 (1.054)	–0.193 (0.978)			
T2	0.141^d^ (0.912)	–0.064^i^ (1.010)	–0.022 (1.002)	–0.083 (1.036)			
Left arm (flex+ext), mean (SD)					0.15 (2, 454)	0.49 (3, 227)	2.78^c^ (6, 454)
T0	–0.046 (0.728)	0.009 (0.826)	0.023 (0.765)	0.029 (0.747)			
T1	0.145^f^^,^^g^ (0.682)	0.031 (0.600)	0.006 (0.750)	–0.155^f^^,^^h^ (0.810)			
T2	0.088 (0.740)	0.097 (0.721)	0.009 (0.817)	–0.098 (0.714)			
Right arm (flex+ext), mean (SD)					0.11 (2, 448)	0.13 (3, 224)	4.29^j^ (6, 448)
T0	–0.168^f^ (0.743)	0.083 (0.646)	0.086^f^ (0.767)	0.024 (0.732)			
T1	0.037^g^ (0.726)	0.088 (0.644)	–0.038 (0.780)	–0.075 (0.708)			
T2	0.115^k^ (0.666)	–0.049 (0.772)	–0.029 (0.836)	–0.087 (0.590)			
Torso rotation left, mean (SD)					0.26 (2, 440)	0.32 (3, 220)	0.77 (6, 440)
T0	–0.145 (1.045)	0.018 (1.114)	0.007 (1.038)	0.082 (0.872)			
T1	–0.064 (0.994)	–0.111 (1.061)	0.023 (1.038)	–0.013 (0.930)			
T2	–0.109 (0.986)	–0.099 (1.088)	0.071 (1.019)	–0.018 (0.930)			
Torso rotation right, mean (SD)					0.27 (2, 440)	0.51 (3, 220)	1.54 (6, 440)
T0	0.063 (1.133)	0.037 (0.929)	–0.078 (0.999)	0.036 (0.874)			
T1	0.189 (1.030)	–0.231 (0.940)	–0.139 (0.982)	0.068 (1.041)			
T2	0.074 (1.094)	0.002 (1.042)	0.018 (1.003)	–0.069 (0.087)			

a Adherers: ≥1.5/wk

b Nonadherers: <1.5/wk

c *P*<.05.

d Differences between T0 and T2 (*P*<.05).

e Differences between T1 and T2 (*P*<.05).

f Differences between groups (*P*<.05).

g Differences between T0 and T1 (*P*<.01).

h Differences between T0 and T1 (*P*<.05).

i Differences between T1 and T2 (*P*=.05).

j *P*<.01.

k Differences between T0 and T2 (*P*<.01).

Concerning arm strength, a group×time interaction was observed for lifting right arm to the side (η^2^=0.034; Table 4). Specific improvements were noted in the Adherers from T0 to T1 (*M*_diff0,1_=0.247, *P*=.02, *d*=0.395) and from T0 to T2 (*M*_diff0,2_=0.213, *P*=.02, *d*=0.327; Table 4). Pairwise differences were observed for arm strength variables, although no interactions were found: the Adherers improved on right arm forward from T0 to T1 (*M*_diff0,1_=0.271, *P*=.006, *d*=0.444) and from T0 to T2 (*M*_diff0,2_=0.308, *P*=.002, *d*=0.466), on left arm forward from T0 to T1 (*M*_diff0,1_=−0.215, *P*=.03, *d*=0.332) and from T0 to T2 (*M*_diff0,2_=−0.266, *P*=.005, *d*=0.399), on left arm to side from T0 to T1 (*M*_diff0,1_=0.201, *P*=.03, *d*=0.331) and from T0 to T2 (*M*_diff0,2_=0.179, *P*=.04, *d*=0.272; Table 4).

No change was observed in lower body strength (sit-to-stand; Table 4).

**Table 4.** *z* scores of T0 (baseline), T1 (4 wk), and T2 (8 wk) strength measures.

	Personalized exercise adherers^a^ (1)	Personalized exercise nonadherers^b^ (2)	General activity (3)	Control (4)	*F* Time (*df*)	*F* Group (*df*)	*F* Interaction (*df*)
Left arm forward, mean (SD)					2.58 (2, 388)	2.17 (3, 194)	1.86 (6, 388)
T0	−0.052^c^ (0.821)	0.448^c^ (0.186)	0.103 (1.108)	−0.037 (0.910)			
T1	−0.267^c^^,^^d^ (0.710)	0.236^c^ (0.943)	0.029 (1.170)	0.107^c^ (0.981)			
T2	−0.320^e^^,^^f^ (0.688)	0.337^e^ (0.971)	−0.045 (1.088)	0.029 (0.912)			
Right arm forward, mean (SD)					0.93 (2, 392)	1.84 (3, 196)	1.92 (6, 392)
T0	0.015 (0.869)	0.300 (1.118)	0.012 (1.048)	0.063 (0.983)			
T1	−0.257^c^^,^^g^ (0.859)	0.308^c^ (0.968)	−0.011 (1.082)	0.105 (0.998)			
T2	−0.293^c^^,^^f^ (0.793)	0.337 (1.062)	−0.033 (1.049)	0.130^a^ (0.950)			
Left arm to side, mean (SD)					0.22 (2, 404)	3.27^h^ (3, 202)	1.47 (6, 404)
T0	−0.095^c^ (0.865)	0.440^c^ (1.271)	−0.059^c^ (0.960)	0.023 (0.931)			
T1	−0.296^c^^,^^d^ (0.778)	0.373^c^ (0.960)	−0.054 (1.122)	0.157^c^ (0.989)			
T2	−0.274^c^^,i^ (0.690)	0.492^c^ (1.081)	−0.085 (1.012)	0.078^c^ (0.959)			
Right arm to side, mean (SD)					0.45 (2, 388)	1.52 (3, 194)	2.26^h^ (6, 388)
T0	0.318 (1.083)	0.318 (1.083)	0.063 (1.134)	−0.060 (0.868)			
T1	−0.253^c^^,^^d^ (0.799)	0.319^c^ (0.916)	−0.001 (1.051)	0.153^c^^,^^d^ (1.045)			
T2	−0.219^c^^,^^i^ (0.817)	0.285^c^ (1.034)	−0.038 (1.016)	0.078 (0.958)			
Total forward (left+right), mean (SD)					2.84 (2, 408)	2.44 (3, 204)	1.88 (6, 408)
T0	−0.001^c^ (0.838)	0.517^c^ (1.182)	0.059 (1.086)	0.052 (0.975)			
T1	−0.228^c^^,^^d^ (0.796)	0.318^c^ (0.910)	0.004 (1.098)	0.150^i^ (1.007)			
T2	−0.275^c^^,^^f^ (0.753)	0.402^c^ (1.047)	−0.275 (0.753)	0.120^c^ (0.973)			
Total to side (left+right), mean (SD)					0.35 (2, 412)	2.23 (3, 206)	1.83 (6, 412)
T0	−0.048 (0.900)	0.379 (0.166)	0.024 (1.069)	−0.019 (0.882)			
T1	−0.277^c^^,^^d^ (0.773)	0.346^c^ (0.930)	−0.028 (1.085)	0.142^c^ (0.997)			
T2	−0.229^c^^,^^i^ (0.776)	0.388^c^ (1.052)	−0.040 (1.020)	0.077 (0.927)			
Total left (forward+side), mean (SD)					1.09 (2, 414)	2.61 (3, 207)	1.93 (6, 414)
T0	−0.072^c^ (0.823)	0.444^c^ (1.160)	0.052 (1.067)	−0.005 (0.893)			
T1	−0.277^c^^,^^d^ (0.726)	0.305^c^ (0.923)	0.007 (1.135)	0.142^c^ (0.960)			
T2	−0.283^c^^,^^i^ (0.686)	0.415^c^ (1.000)	−0.029 (1.043)	0.069^c^ (0.923)			
Total right (forward+side), mean (SD)					0.38 (2, 442)	3.91^j^ (3, 221)	1.88 (6, 442)
T0	−0.153^c^ (0.807)	0.512^c^ (1.004)	0.041^c^ (0.972)	−0.043^c^ (0.938)			
T1	−0.256^e^^,^^d^ (0.775)	0.471^e^ (0.888)	0.040 (1.006)	0.029 (1.000)			
T2	−0.237^e^ (0.771)	0.591^e^ (1.011)	0.037 (0.995)	−0.026 (0.988)			
Lower body strength (sit to stand), mean (SD)					1.10 (2, 420)	2.16 (3, 210)	0.72 (6, 420)
T0	−0.158 (0.974)	0.220 (0.891)	0.099 (1.048)	−0.099 (0.976)			
T1	−0.254a^c^ (0.965)	0.325a^c^ (1.121)	0.126^c^ (1.043)	−0.025 (0.910)			
T2	−0.281 (0.877)	0.077 (0.947)	0.115 (1.069)	−0.061 (0.923)			

a Adherers: ≥1.5/wk

b Nonadherers: <1.5/wk

c Differences between groups (*P*<.05).

d Differences between T0 and T1 (*P*<.05).

e Differences between groups (*P*<.01).

f Differences between T0 and T2 (*P*<.01).

g Differences between T0 and T1 (*P*<.01).

h *P*<.05.

i Differences between T0 and T2 (*P*<.05).

j *P*<.01.

### Follow-Up

No group×time interaction was observed on any of the outcomes. Significant (*P*<.05) time effects were revealed, indicating significant deterioration in both groups—the personalized Adherers and the general groups—during the 4-week follow-up.

## Discussion

### Principal Findings

We examined the hypothesis that remotely delivered, personalized multicomponent exercise for older adults—based on a simple yet reliable and accurate smartphone motor fitness assessment and individually tailored using machine learning—can improve balance, flexibility, and strength among older adults, all without the need for a laboratory or professional supervision.

The results of this randomized controlled study demonstrated that a multicomponent personalized exercise program among older people was more effective when compared to active controls who were counseled to perform regular exercise according to WHO guidelines, as well as a passive control group (who received no intervention). Furthermore, improvements were achieved with as few as 1.5 tailored exercise sessions per week over 8 weeks and in many fitness measurements, even within 4 weeks.

The study tool has been refined from the initial prototype and proof of concept [14-16] and in its present form was proven to be sufficiently sensitive to measure both improvement and decline across the motor fitness measures, in as few as 4 weeks following the initiation or cessation of the exercise intervention, respectively.

The improvement in balance is of particular importance, as the relationship between balance impairment and falls [31] and the positive effect of exercise on balance and fall prevention among older people is well documented [18]. However, exercise interventions typically involve group sessions in neighborhood clubs or physical therapy laboratories [32], requiring a specialized trainer or therapist, and mostly target participants at risk of falls [32,33]. Balance control is multifactorial [17,34], and the novelty of the study tool presented in this study is not only its potential widespread application but also the fact that it includes 4 static and 2 dynamic balance tests, each comprising 5 DMs assessing body sway in all directions (mediolateral, anterior-posterior, and superior-inferior), offering a complete overview of the participant’s balance. Simultaneously, the balance-focused segment of our exercise programs addresses all components of balance, encompassing 5 subcategories: static, dynamic, vestibular, leg strength, and leg flexibility [35].

Flexibility is a fitness component known to typically decline with advancing age, presenting a major contribution to reduced functioning [19]. Although amenable to improvement through focused exercise [20], nonetheless, current guidelines provide only minimal detailed attention, and consequently, this element of fitness often remains neglected [9,10]. Our findings revealed significant improvement in flexibility in as little as 4 weeks of tailored exercise for upper body (arm) flexibility (both extension and flexion), which was most pronounced among the personal adherers, while measures of torso rotation remained unchanged. In all likelihood, this lack of observed change in torso flexibility is an artifact due to the technical difficulty measuring this vector with the smartphone.

The importance of upper body strength is notable with advancing age, with weakness associated with painful symptomatology [36], upper limb dysfunction, and decreased performance measures [21]. It should be noted that upper extremity strength can be represented by several measurements, such as the widely used arm curl test [37,38]. We chose to focus on the shoulder joint because age-related decline in maximal upper extremity torque is greater at the shoulder than at the elbow and wrist joints [39].

In contrast to the shoulder, we did not observe significant differences in leg strength, although a trend for improvement was seen in the intervention group. In our pilot study, we previously observed improvements in leg strength using sit-to-stand repetitions over a 30-second period [14,15]. However, to simplify testing, we modified this measure to a time score for 10 repetitions, which led to a ceiling effect. Importantly, leg strength is essential for functioning in older adults [40,41], and evaluating the impact of exercise on this component is crucial. Since the sit-to-stand repetitions (as many repetitions as possible over 30 s) were shown to be sensitive to changes following exercise, we plan to return to this method in future assessments.

Our study is unique in using a simple yet accurate smartphone platform to incorporate a remote assessment of balance, flexibility, and strength to serve as the baseline for a subsequent matched range of exercises, spanning these modalities of motor fitness. The existing models rely heavily upon fitness trackers, built-in accelerometers, and on occasion GPS to remotely monitor steps, endurance, and energy consumption. However, the critical elements of balance, flexibility, and shoulder strength are neglected, despite their crucial importance among older people. It is worth mentioning that recent evidence is emerging from the rehabilitation setting, supporting the use of smartphones for assessing balance or flexibility (range of motion) in older adults; however, mostly for highly specific conditions requiring clinical supervision [42-45].

Notably, while the platform demonstrated sufficient sensitivity to detect changes at a low threshold (1.5 sessions per week over 4 weeks), it remains uncertain whether this frequency is adequate to produce meaningful clinical health benefits. This concern is further emphasized by the small effect sizes observed—despite their statistical significance—and by the higher frequency recommended in WHO guidelines. Based on our findings, we cannot assert that this level of activity is sufficient for inducing clinical health improvements.

Similarly, the deterioration observed after the 4-week follow-up period without exercise was also subtle, suggesting that short-term inactivity may not necessarily lead to clinically relevant declines in fitness. This aligns with the broader detraining literature, which indicates that the rate of fitness loss is highly dependent on the initial training volume—encompassing factors such as intensity, duration, and frequency. Individuals with a higher training volume tend to experience a more gradual decline in fitness, whereas those with lower initial levels may exhibit more rapid losses [46-48].

In our study, the smartphone app was able to detect subtle yet significant improvements in fitness after 4 weeks of exercise, which then disappeared following 4 weeks of inactivity. While neither of these changes is likely to translate into meaningful clinical health effects, they underscore the application’s sensitivity in capturing even small fluctuations in fitness over time—both in improvement and decline. This capability highlights its potential as a tool for remotely monitoring fitness trends that might otherwise go unnoticed outside of laboratory settings.

An interesting finding of our study is the notable relationship between participants’ habitual physical activity and sedentary time before starting the intervention and their subsequent adherence to the exercise regimen. Significant differences were observed between adherers and nonadherers, with adherers engaging in more habitual physical activity and nonadherers spending more time sedentary. It is important to note that the classification into adherers and nonadherers was made post facto, only after the intervention ended. This suggests that nonadherence to physical activity, often associated with a more sedentary lifestyle, may reflect an underlying behavioral predisposition that persists even when individuals voluntarily enroll in an exercise program.

### Study Limitations

Several limitations to our study deserve mention. First, the rate of adherence to the exercise program was accurately measured via the smartphone among the interventional group, while we had to rely upon self-reported data among the controls. Attempts to categorize the active control group into adherers and nonadherers based on self-reports, using the same cutoff points as described for the personalized group, showed no clear trends. To address this limitation, we compared the overall percentage of improvers in the intervention group to those in the active control group over the 8-week program, irrespective of the degree of adherence. Compared to the controls, the intervention participants showed higher rates of improvement for static balance (46% vs 33.3%, *P*=.07), dynamic balance (53.6% vs. 28.4%, *P*=.002), arm flexion (52.8% vs 43.4%, *P*=.48), and arm extension (46.1% vs 34.6%, *P*=.30).

Second, in the current study, in order to generate accurate data for the study, the trained study personnel performed the testing of participants. However, the ultimate goal of the application is that participants will be able to assess themselves (or possibly with the assistance of a lay person, eg, family member).

Another limitation is the sample’s bias toward participants from higher socioeconomic status. As this is a randomized controlled study, this bias does not affect the main outcome—the improvement of the intervention group over the controls. However, it may impact the generalizability of the study results to other populations.

In addition, in the current study, we applied only 2 levels of difficulty for each of the 3 movement components. As large-scale data accumulate, there is potential to expand and further define more variations of movement profiles.

Furthermore, physical fitness may include more components than the ones included in our study. For example, for assessing strength, while the selected tests did represent the major muscle groups, nonetheless, not all muscle groups were included. We also did not include other components such as eye–hand coordination or fine motor abilities. However, we selected the most appropriate tests for assessing motor and physical fitness in old age, taking into consideration medical aspects and safety precautions (for more information on designing the tests, see [14]).

The interaction with the technology—the smartphone—may also be problematic. For example, using the smartphone screen was inconvenient for some participants, and larger screens would improve the ease of performing the exercises. This could be solved by connecting the smartphone to a large screen. In addition, the videoed exercise program was running in a continuous manner on the phone screen; however, if the participant wanted to repeat an exercise before moving to the next one, or to stop for some reason, he or she had to change his or her current position, approach the phone, stop the program, and restart it again.

It may be argued against the smartphone sensors that there are settings where more accurate assessments may give more accurate results. Although applicable for research, these settings are inaccessible to the general population as they are scarce and expensive to use. Prior work [16,49] has already demonstrated that smartphones offer a reliable tracking of motor functions and therefore offer a sufficient, even if not perfect, means for evaluation in large scales.

The study sample was generally healthy at the time of enrollment, as assessed by the study physician. Enrolled participants were cognitively intact, free from any hospitalization in the last year for any cardiac or neurological reason, independent in daily function, able to walk without assistance, and with a low fall risk. Furthermore, we included a 41-item frailty assessment (see Table 1), which confirmed very low levels of frailty across all participants. Although comorbidities or other current health conditions were not formally assessed, nonetheless, the study sample’s health profile of high cognitive function, independent functional and mobility status, very low frailty levels, and no recent cardiological or neurological illness for hospitalization was suggestive of an overall health status.

### Using Artificial Intelligence for Extending and Refining the Tailored Exercise Program

Generating optimal personalized exercise program should rely not only on assessing the level of fitness but also considering basic aspects of the individual health status and behavior patterns. In line with personalized genomics and big data accumulation, a possible future upgraded version of this application might be used in conjunction with data drawn from genetic, molecular, clinical, social, and behavioral domains in order to achieve an optimal prescription of personalized exercise programs.

Furthermore, large-scale data will enable an in-depth analysis of individual movement capacities, a more accurate assessment of a wide range of fitness and mobility parameters, and, subsequently, more specific personalized exercise programs. For instance, when assessing balance in individuals with stability issues, it is beneficial to analyze the specific directions of sway—mediolateral, anteroposterior, and superior-inferior. Similarly, an arm strength test can be broken down into separate actions of “lifting the hand up” and “lowering it down,” while the sit-to-stand test can be divided into “getting up” and “sitting down.” Accurately localizing movement impairments has important clinical implications.

The potential ramifications of this study are multifold. First, we provide a model whereby a home-based, highly detailed remote analysis of fitness and mobility parameters provides objective quantitative data as a basis for subsequent tailored exercise interventions at the individual level. Furthermore, the sensitivity of the remote sensors was shown to be sufficient to enable an adaptive, precise, and updating exercise program, determined by the participants’ progress over time. In addition, the continually updating database serves to constantly improve the machine learning precision of decisions concerning the selection of appropriate exercises. Second, our study included only healthy, independent adults aged 65 years and over. We provide evidence to support a platform that has the potential to be adapted to meet the specific needs and abilities of various healthy and patient populations. Specifically, among older people, it is plausible that targeted and individualized exercise interventions are likely to play a beneficial role in improving intrinsic capacity and resilience, moderating frailty and deconditioning. Similarly, individualized programs can be further refined to address specific needs of populations with mobility limitations, such as those at risk of falls or confined to wheelchairs, as well as the potential applicability among participants with cognitive impairments.

### Summary and Conclusions

In this randomized controlled study, we examined a novel home-based approach for personalized exercise programs for older adults, utilizing a simple smartphone that obviates the need for a laboratory or professional intervention. Through smartphone accelerometer and gyroscope sensors, we remotely assessed key components of motor fitness, including balance, flexibility, and strength. Based on these assessments, a machine-learning-generated personalized exercise program, tailored to each individual’s needs, was delivered via video directly on the smartphone. The results demonstrated that a multicomponent personalized exercise program was more effective when compared to active controls who were counseled to perform regular exercise according to WHO guidelines, as well as a passive control group who received no intervention. Furthermore, improvements were achieved with as few as 1.5 tailored exercise sessions per week over 8 weeks and in many fitness measurements, even within 4 weeks. The remote delivery of user-friendly multicomponent exercise programs to older people has the potential for widespread health benefits. Given the potential of this approach to be extended to various groups of older adults, including those with mobility and cognitive impairments, and considering the advantages of cost-free digital technology (assuming widespread smartphone ownership), health care providers may adopt this approach to provide home-based strategies for health promotion.

## Supplementary material

10.2196/73145 Multimedia Appendix 1 Values of cutoff point of adherers and nonadherers.

10.2196/73145 Multimedia Appendix 2 Attrition participation rate.

10.2196/73145 Checklist 1 CONSORT-eHEALTH checklist (V 1.6.1).

## Abbreviations:

DM: digital marker
WHO: World Health Organization
