# Supplementary material for: A Smartphone Platform for Remote Motor Fitness Assessment and AI-Generated Personalized Exercise Programs for Older Adults: Randomized Controlled Trial
Source: J Med Internet Res. 2025 Oct 15;27:e73145. doi: 10.2196/73145 (PMC12527324; doi:10.2196/73145)
Supplement: Multimedia Appendix 1 [file jmir-v27-e73145-s001.pdf]

	**1.5 X/wk**<br>**P value**<br>**AD: N = 66**<br>**NAD: N = 26**	**2 X/wk**<br>**P value**<br>**AD: N = 60**<br>**NAD: N = 32**	**2.45 X/wk**<br>**P value**<br>**AD: N = 46**<br>**NAD: N = 46**	**3 X/wk**<br>**P value**<br>**AD: N = 41**<br>**NAD: N = 51**
Balance: Leg Stance	0.782	0.661	0.853	0.924
Balance: Tandem Stance	0.051 (AD↑C,G↓)	0.044 (AD↑C,G↓)	0.070	0.075
Static Balance	0.170	0.145	0.170	0.241
Dynamic Balance	0.004 (AD↑C,G↓)	0.002 (AD↑C↓)	0.009 (AD↑C↓)	0.003 (AD↑C↓)
Balance Total	0.006 (AD↑NAD,G↓)	0.003 (AD↑NAD,C,G↓)	0.029 (AD↑C,G↓)	0.036 (AD↑G↓)
Flexibility: Arm Extension	0.012 (AD↑C↓)	0.008 (AD↑C,G↓)	0.013 (AD↑C,G↓)	0.021 (AD↑C↓)
Flexibility: Arm Flexion	0.000 (AD↑C,G↓)	0.000 (AD↑C,G↓)	0.000 (AD↑C,G↓)	0.000 (AD↑C,G↓)
Flexibility: Arm Left	0.011 (AD↑C↓)	0.010 (AD↑C↓)	0.008 (AD↑C↓)	0.007 (AD↑C↓)
Flexibility: Arm Right	0.000 (AD↑C,G↓)	0.000 (AD↑C,G↓)	0.001 (AD↑C,G↓)	0.000 (AD↑C,G↓)
Strength: Arm Forward	0.083	0.118	0.079	0.072
Strength: Arm to the Side	0.091	0.150	0.166	0.148
Strength: Arm Total Left	0.075	0.120	0.118	0.095
Strength: Arm Total Right	0.082	0.029 (AD,NAD↑C↓)	0.259	0.210
Strength: Arm All	0.028 (AD↑C↓)	0.144	0.099	0.024 (AD↑C↓)

AD = Adherers, NAD = non-Adherers, G = General activity, C = Control

Multimedia Appendix 1: Adherers - non-Adherers potential cut-off points (p values of time*group interactions)
