# Supplementary material for: A Smartphone Platform for Remote Motor Fitness Assessment and AI-Generated Personalized Exercise Programs for Older Adults: Randomized Controlled Trial
Source: J Med Internet Res. 2025 Oct 15;27:e73145. doi: 10.2196/73145 (PMC12527324; doi:10.2196/73145)
Supplement: Multimedia Appendix 2 [file jmir-v27-e73145-s002.pdf]

Group	n Baseline	Arm Flexion n	Arm Flexion %	Arm Extension n	Arm Extension %	Arm Strength n	Arm Strength %	Balance Total n	Balance Total %	Dynamic Balance n	Dynamic Balance %	Static Balance n	Static Balance %
Adherers	66	63	**95.45**	63	**95.45**	61	**92.42**	63	**95.45**	61	**92.42**	61	**92.42**
non-Adherers	26	24	**92.31**	24	**92.31**	23	**88.46**	23	**88.46**	21	**80.77**	23	**88.46**
General	80	75	**93.75**	77	**96.25**	75	**93.75**	73	**91.25**	66	**82.50**	72	**90.00**
Control	67	66	**98.51**	65	**97.01**	57	**85.07**	61	**91.04**	58	**86.57**	61	**91.04**

Multimedia appendix 2: Rate of Attrition
